# First follow‐up radiographic response is one of the predictors of local tumor progression and radiation necrosis after stereotactic radiosurgery for brain metastases

**DOI:** 10.1002/cam4.1149

**Published:** 2017-08-04

**Authors:** Mayur Sharma, Xuefei Jia, Manmeet Ahluwalia, Gene H. Barnett, Michael A. Vogelbaum, Samuel T. Chao, John H. Suh, Erin S. Murphy, Jennifer S. Yu, Lilyana Angelov, Alireza M. Mohammadi

**Affiliations:** ^1^ Department of Neurosurgery The Rose Ella Burkhardt Brain Tumor and Neuro‐Oncology Center Neurological Institute Cleveland Clinic 9500 Euclid Avenue, CA‐50 Cleveland Ohio 44195; ^2^ Department of Biostatistics Cleveland Clinic Cleveland Ohio 44195; ^3^ Cleveland Clinic Department of Radiation Oncology 9500 Euclid Avenue, CA‐50 Cleveland Ohio 44195

**Keywords:** Brain metastasis, gamma knife radiosurgery, local progression, tumor

## Abstract

Local progression (LP) and radiation necrosis (RN) occur in >20% of cases following stereotactic radiosurgery (SRS) for brain metastases (BM). Expected outcomes following SRS for BM include tumor control/shrinkage, local progression and radiation necrosis. 1427 patients with 4283 BM lesions were treated using SRS at Cleveland Clinic from 2000 to 2012. Clinical, imaging and radiosurgery data were collected from the database. Local tumor progression and RN were the primary end points and correlated with patient and tumor‐related variables. 5.7% of lesions developed radiographic RN and 3.6% showed local progression at 6 months. Absence of new extracranial metastasis (*P* < 0.001), response to SRS at first follow‐up scan (local progression versus stable size (*P* < 0.001), partial resolution versus complete resolution at first follow up [*P* = 0.009]), prior SRS to the same lesion (*P* < 0.001), IDL% (≤55; *P* < 0.001), maximum tumor diameter (>0.9 cm; *P* < 0.001) and MD/PD gradient index (≤1.8, *P* < 0.001) were independent predictors of high risk of local tumor progression. Absence of systemic metastases (*P* = 0.029), good neurological function at 1st follow‐up (*P *≤ 0.001), no prior SRS to other lesion (*P* = 0.024), low conformity index (≤1.9) (*P* = 0.009), large maximum target diameter (>0.9 cm) (*P* = 0.003) and response to SRS (tumor progression *vs*. stable size following SRS [*P* < 0.001]) were independent predictors of high risk of radiographic RN. Complete tumor response at first follow‐up, maximum tumor diameter <0.9 cm, tumor volume <2.4 cc and no prior SRS to the index lesion are good prognostic factors with reduced risk of LP following SRS. Complete tumor response to SRS, poor neurological function at first follow‐up, prior SRS to other lesions and high conformity index are favorable factors for not developing RN. Stable or partial response at first follow‐up after SRS have same impact on local progression and RN compared to those with complete resolution or progression.

## Introduction

Brain metastasis (BM) is the most common brain tumor leading to significant morbidity and mortality in patients with systemic malignancies. The median overall survival in patients with BM ranges between 5.7 and 15.2 months, based on the intervention 1, 2, 3. BM affects up to 40% of patients with a range of systemic malignancies and an incidence ranging between 8.3 and 14.3/100,000 populations based on epidemiological studies 3, 4, 5, 6, 7, 8, 9, 10. With the increase in the incidence of systemic malignancies coupled with advances in systemic treatment regimens, BMs pose significant challenges to physicians managing these patients.

Stereotactic radiosurgery (SRS) is a well‐established treatment modality (either alone or in combination with surgery/WBRT) in managing patients with single or multiple BMs with improved local control rates and minimal side effects 1, 11, 12, 13, 14, 15, 16, 17. Also, SRS alone has been shown to have lesser cognitive decline with no difference in overall survival compared to SRS and WBRT at 3 months in patients with 1–3 BMs 18. SRS has been reported to have a local tumor control rate (at 1 year) of 80–90% as an upfront therapy 14, 19, 20.

Despite good local tumor control, dose escalation is often limited by adverse radiation effects (AREs), particularly radiation necrosis (RN) with an incidence ranging between 5% and 24% 21, 22, 23, 24, 25. Also, larger tumor size and volume are associated with increased risk of RN 21, 24, 26, 27. RN is often a clinical challenge due to ambiguity in diagnosis and prolonged treatment regimens that lead to an increase in the neurological morbidity and associated financial burden 22, 28, 29, 30, 31, 32, 33, 34, 35, 36. There is no prior studies addressing the association between first MRI response following SRS and local tumor progression versus RN. Given that local tumor progression and RN following SRS are potential adverse events leading to an increase in the overall morbidity and mortality, we evaluated the role of first follow‐up radiographic response in predicting local tumor progression and RN. We also aimed to identify various patient and tumor‐related factors associated with local progression and RN in a large series of more than 1000 patients with greater than 4000 BMs.

## Materials and Methods

### Clinical data and inclusion/exclusion criterion

Following institutional review board (IRB) approval at the Cleveland Clinic, data of patients who underwent SRS for BM at our center from 2000 and 2012, were retrospectively reviewed. Inclusion criterion for this study were patients >18 years of age who underwent SRS for BM (any size) at our center and exclusion criterion were absence of demographic, imaging or follow‐up data. Therefore, 1427 patients treated at our center during the study period with 4283 BM were included in the analysis. 279 patients (24.77%) had SRS treatment more than once (median 2, range [2, 7]) leading to a total radiosurgical treatment number of 1555 in 1126 patients. Patient demographics (age/gender, systemic cancer, Karnofsky Performance status [KPS], number of brain metastases, presence of extra‐cranial metastases, Graded Prognostic Assessment [GPA], Recursive Partition Analysis [RPA], ECOG [Eastern Cooperative Oncology Group] performance score, neurological status, prior SRS, WBRT, surgical resection, targeted systemic therapy), imaging variables, radiosurgical variables (PD, prescription dose), maximum dose, conformity index, heterogeneity index, gradient index] tumor variables (location, laterality, number, maximum diameter, volume) and follow‐up data were retrospectively collected from the electronic medical records.

### Gamma knife radiosurgery procedure and clinical follow‐up

Gamma Knife radiosurgery (SRS) was performed using models B, C, 4C, and Perfexion, (Elekta Instruments AB, Stockholm, Sweden) during the study period, following informed and valid consent. Briefly, a volumetric MRI (T1 MPRAGE with contrast; Siemens AG, Erlangen, Germany) was obtained on the day of procedure either prior to or following Leksell (Elekta AB, Stockholm, Sweden) frame placement. A stereotactic CT scan was then obtained following frame placement. The CT and MRI scans were transferred to the planning station and tumor volumes along with risk areas were defined. Thereafter a conformal plan was created, using a combination of shots. A 201 or 192 Gamma Knife radiation source unit was used to deliver the radiation and patient was discharged the same day. The Radiation Therapy Oncology Group (RTOG) 90–05 guidelines were used as a reference to prescribe the radiation dose to the tumor margins based on the largest diameter of the lesion (≤20 mm = 24 Gy, 21–30 mm = 18 Gy and 31–40 mm= 15 Gy)^37^. Patients were followed with clinical examinations and MRIs [T1 MPRAGE and FLAIR (Fluid‐attenuated inversion recovery)] at 4–6 weeks after SRS and 3 months thereafter. Clinical or imaging follow‐up was tailored according to the individual requirements. SRS treatment was defined as upfront (SRS was used as a first line treatment for BM), salvage (SRS was used following failure of other therapies for BM) and boost SRS (SRS for lesions which were treated with WBRT before with no evidence of local progression between two treatment modalities).

### End points

Local tumor progression and radiographic RN were the end points in this study. These end points were correlated with tumor‐related variables and radiographic response during follow‐up. Radiographic response to SRS during follow‐up was defined as per RANO guidelines (complete response, partial response, stable and progression) 38. All treated lesions were measured in longest dimension on postprocedure MRIs and were compared to the pre‐SRS MRI scans to assess the response 38. Tumor progression was defined as ≥20% increase in the sum of longest diameters of target lesions 38.

Radiographic RN was defined based on the radiological or, rarely, pathological tissue. Additional imaging modalities such as MR perfusion scan, FDG‐ PET scan or short‐term follow‐up MRI scans (1–3 months) were used to differentiate between RN and local tumor progression in doubtful cases 22. In addition, we also used the recommendations of our multidisciplinary brain tumor board for the diagnosis in patients with conflicting imaging results. Tumor variables and response following SRS were analyzed in prognosticating the risk of local tumor progression and radiographic RN. The median radiographic follow‐up of the lesions was 12.3 months.

### Statistical methods

Statistical analysis was performed using SAS 9.4 (SAS Institute, Inc., Cary, NC. Categorical data were analyzed as frequency counts and percentages; whereas, measured data were evaluated using medians and ranges. Time to event data was summarized using the cumulative incidence estimates. Local progression was treated as an event and deaths were treated as competing risks (CK). Local tumor progression was calculated from the date of the SRS procedure. Univariate and multivariate analysis were performed on a per‐lesion basis using marginal competing risk models and marginal logistic regression for local tumor progression and RN, respectively, which were adjusted by the patient and SRS treatment. Tumor variables showing significant association with these end points on univariate analysis were included in a multivariate model. Stepwise variable selection with *P* = 0.05 was used as the criteria for entry and retention in the model to compute independent prognostic factors. Once the final prognostic models were defined, prognostic indexes were created by assigning weights to the levels of each factor that were proportional to the corresponding regression coefficients and then summed the individual weights for each patient. Using these sums, prognostic groups were created as per Cox's suggestion 39 and prognostic group outcomes were then analyzed.

## Results

### Patient demographics and lesion characteristics

Overall, 47.4% (676/1427) of patients were male, and median age at the time of SRS was 60.3 years. Less than half of the patients (40.7%, *n* = 580) had multiple systemic metastases and 24% (*n* = 342) had no systemic metastases at SRS. At the time of SRS, 64.3% (*n* = 909) of the patients had systemic progression, and 50.3% (*n* = 689) had new extra‐cranial metastases (ECM) at SRS. A majority of patients had good performance status at SRS [76.4%, *n* = 1087 had KPS ≥ 80]. However, 76.6% (*n* = 1219) of patients were classified as poor prognostic group by GPA criteria (scores < 3). 45.8% (*n* = 647) of patients had received chemotherapy for systemic disease within a month of first SRS. The median number of radiosurgical targets was 2 (range 1, 17); 42.5% (*n* = 606) of patients had 2–4 targets and 11% had 5–10 targets for SRS. More than half of the patients (54.1%, *n* = 772) had lesions < 2 cm and 18.2% (*n* = 260) had lesions > 3 cm in size. Of note, 5.5% (*n* = 78) of patients had brainstem metastases. More than half of the patients (56.8%, *n* = 809) had upfront SRS, 23.3% (*n* = 332) had salvage SRS and 1.9% (*n* = 284) had boost SRS. Also, 68.7% (*n* = 986) of patients had neurologic impairment prior to SRS (mild = 49%, *n* = 705; moderate = 17.2%, *n* = 245 and severe = 2.5%, *n* = 36). A minority of patients had hemorrhage prior to SRS (5.8%, *n* = 82) and following SRS (6.0%, *n* = 69). Only four patients (five lesions) with post‐SRS hemorrhage had hemorrhage prior to SRS on MRI.

A total of 1555 SRS treatments were performed for 4283 lesions during the study period. The median tumor volume and median maximum tumor diameter was 0.24 cc and 0.90 cm, respectively. Tumors were treated to a median prescription IDL of 53.5% (range: 50–95). 30.3% (*n* = 1298) of lesions had prior SRS to another lesion. Median PD, IDL%, conformity, maximum dose, and MD/PD gradient index were 24 Gy, 55%, 1.9, 40.1, and 1.8, respectively. 55.4% (*n* = 2373) of lesions had prior WBRT, 18.3% (*n* = 783) had prior surgery and 0.68% (*n* = 29) had prior SRS to the same lesions. 27.0% (*n* = 1157) of the lesions were radioresistant histologies and resection of nonindex lesion (83.6%, *n* = 635) was the most commonly performed surgical procedure.

In terms of response, more than half of the patients (57.5%, *n* = 659) had partial response, 5.6% (*n* = 64) had complete response, 8.8% (*n* = 101) showed progression on first follow‐up MRI and 36% (*n* = 410) showed progression on second follow‐up MRI. In addition, 57% of patients (*n* = 648) had neurologic impairment (mild = 41.7%; moderate = 12.6% and severe = 2.7%) at first follow up following SRS compared to 69.2% of patients (mild = 49.5%; moderate = 17.2% and severe = 2.5%) at SRS. 11.6% of the patients had new neurological deficit at first follow up following SRS (Table 1).

**Table 1:** Patient characteristics (per patient) (statistics presented as median (min, max) or N [column %])

Factor	Total (*N* = 1427)
Age	60.3 (16.8,92.7)
Gender (female)	751 (52.6)
Systemic metastases^a^	
Single	503 (35.3)
Multiple	580 (40.7)
Systemic progression at SRS^a^ (Yes)	909 (64.3)
New ECM metastases at SRS^a^(Yes)	689 (50.3)
Last month chemotherapy^a^ (Yes)	647 (45.8)
GPA group a	
A	317 (22.3)
B	902 (63.4)
C	144 (10.1)
D	59 (4.1)
KPS^a^	
60 or less	111 (7.7)
70	227 (15.9)
80	424 (29.8)
90	597 (41.9)
100	66 (4.6)
Neurological symptoms	
Mild	705 (49.5)
Moderate	245 (17.2)
Severe	36 (2.5)
Type of SRS^a^	
Upfront	809 (56.8)
Salvage	332 (23.3)
Boost	284 (19.9)
Brainstem involvement (yes)	78 (5.5)
Total intracranial volume a	2.6 (0.03,83.0)
Number of targets per SRS a	2.0 (1.00,17.0)
1	642 (45.0)
2–4	606 (42.5)
5–10	168 (11.7)
>10	11 (0.8)
Target lesion size	
< 2 cm	772 (54.1)
2–3 cm	395 (27.7)
>3 cm	260 (18.2)
Mean iso‐dose line a	53.5 (50.0,95.0)
1st f/u new ND a (Yes)	133 (11.6)
1st f/u Neurological symptoms	
Mild	474 (41.7)
Moderate	143 (12.6)
Severe	31 (2.7)
Post–SRS hemorrhage a (Yes)	69 (6.0)
Response to SRS a	
Complete resolution	64 (5.6)
Partial resolution	659 (57.5)
Stable	323 (28.2)
Progression	101 (8.8)

a Data not available for all subjects. Missing values: systemic met = 2, systemic progression at SRS = 14, enlargement at SRS = 57, last month chemo = 14, recursive partition analysis = 4, GPA = 6, GPA group = 5, KPS = 2, Neurological symptoms = 441, pre‐SRS hemorrhage = 4, SRS type = 2, number of targets per SRS = 3, 1st f/u Neurological symptoms = 291, 1st f/u ND = 281, total intracranial volume = 3, number of lesions <2 cm = 1, number of lesions >3 cm = 4, Mean iso‐dose line = 46, 1st f/u KPS = 280, post‐SRS hemorrhage = 281, Enlargement after SRS = 289, Response to SRS = 280, pathology = 3.

### Univariate and multivariate analysis for local progression

Univariate analysis based on per lesion level and adjusted by patient, and SRS treatment revealed 10 patient‐related variables (young age, absence of systemic metastasis, absence of systemic disease progression at SRS, absence of new extracranial metastasis at SRS, good neurological function at SRS, presence of post‐SRS hemorrhage, stable or tumor progression following SRS) and 10 lesion‐related variables (no prior SRS to the other lesion, prior SRS to the same lesion, large volume of target lesion, large maximum tumor diameter at SRS, low prescription dose, low maximum dose, low prescription IDL, low conformity, and MD/PD gradient index), which were found to be associated with increased risk of local progression (Table 2).

**Table 2:** Univariate analysis for LP and RN (Per lesion analysis adjusted by the patient and SRS treatment)

Factors	Hazard Ratio for LP (95% CI)	*P*	Odds Ratio for RN (95% CI)	*P*
Age	0.99 (0.97,1.00)	*0.046*	0.99 (0.97,1.00)	0.088
Gender (Female vs. male)	0.78 (0.58,1.05)	0.096	0.85 (0.62,1.17)	0.33
Systemic metastasis (no vs. yes)	0.54 (0.38,0.76)	*<0.001*	0.63 (0.43,0.91)	*0.014*
New systemic progression at SRS (no vs. yes)	0.55 (0.41,0.74)	***<0.001***	0.59 (0.43,0.80)	***<0.001***
New ECM at SRS (no vs. yes)	0.44 (0.32,0.60)	***<0.001***	0.79 (0.57,1.08)	0.14
Last month Chemo (no vs. yes)	0.69 (0.36,1.30)	0.25	1.14 (0.64,2.04)	0.65
Neurological symptoms (none vs. mild vs. moderate, severe)	0.81 (0.67,0.99)	***0.036***	0.56 (0.45,0.70)	***<0.001***
SRS Type (Other vs. Boost)	0.80 (0.54,1.18)	0.26	1.11 (0.74,1.68)	0.61
1st f/u Neurological symptoms	0.94 (0.76,1.17)	0.61	0.51 (0.40,0.65)	***<0.001***
Post‐SRS hemorrhage (No vs. Yes)	3.32 (1.79,6.14)	***<0.001***	1.52 (0.63,3.69)	0.35
Response to SRS				
CR vs. PR	0.02 (0.01,0.04)	***<0.001***	10.8 (1.9, 23.7)	***<0.001***
PR vs. Stable	1.25 (0.88,1.78)	0.21	1.03 (0.71, 1.48)	0.88
Stable vs. progression	9.48 (6.01,14.9)	***<0.001***	7.6 (4.48, 12.9)	***<0.001***
Pathology (radio resistance vs. sensitive)	1.13 (0.82,1.54)	0.46	0.99 (0.69,1.40)	0.94
Prior WBRT	0.92 (0.69,1.23)	0.56	0.82 (0.60,1.13)	0.23
Prior SRS to the other lesion (No vs. Yes)	0.68 (0.48,0.97)	***0.031***	0.50 (0.34,0.75)	***<0.001***
Prior SRS to the same lesion (No vs. Yes)^a^	22.98 (16.8,31.4)	***<0.001***	–	–
Volume (cc) (≤2.4 vs. >2.4)	3.19 (2.43,4.19)	***<0.001***	1.94 (1.36,2.78)	***<0.001***
Max dim dimension (≤0.9 vs. >0.9)	4.00 (2.94,5.43)	***<0.001***	3.27 (2.39,4.47)	***<0.001***
PD (≤22 vs. >22)	0.41 (0.31,0.54)	***<0.001***	0.81 (0.59,1.12)	0.20
IDL%(con)	0.96 (0.95,0.98)	***<0.001***	0.97 (0.96,0.99)	***<0.001***
IDL% (≤55 vs. >55)	0.55 (0.42,0.73)	***<0.001***	0.44 (0.33,0.61)	***<0.001***
Conformity (≤1.9 vs. >1.9)	0.60 (0.45,0.80)	***<0.001***	0.42 (0.31,0.57)	***<0.001***
Max dose(con)	0.97 (0.96,0.99)	***0.002***	1.03 (1.00,1.05)	***0.017***
Max dose (≤cut off vs. >cut off)^b^	0.69 (0.53,0.89)	***0.004***	1.58 (1.17, 2.13)	***0.0029***
MD/PD index (con)	3.12 (1.71,5.71)	***<0.001***	4.40 (2.33,8.31)	***<0.001***
MD/PD index (≤1.8 vs. >1.8)	1.66 (1.26,2.18)	***<0.001***	2.23 (1.64,3.04)	***<0.001***

SRS, stereotactic radiosurgery; LP, local tumor progression; RN, radiation necrosis; ECM, Extra cranial metastases; CR, complete resolution; PR, partial resolution; WBRT, whole‐brain radiation therapy; PD, Prescription dose; IDL, Isodose line; MD/PD, Mean dose/prescription dose.

a One of the categories didn't have any event, therefore can't get the odds ratio.

b Max dose of 40 was used as the cutoff for local progression and cutoff of 45 was used for RN.

values in italics means significant , *p*<0.05

On multivariate analysis, of these variables, partial resolution of lesion at first follow‐up MRI compared to complete resolution (HR: 2.20, *P* = 0.009), progression of tumor compared to stable size in first follow‐up MRI following SRS (HR: 10.2, *P* < 0.001) absence of new extra‐cranial metastasis (HR: 0.47, *P* < 0.001), prior SRS to the same lesion (HR:11.96, *P* < 0.001), maximum tumor diameter (> 0.9 cm) (HR: 2.31, *P* < 0.001), IDL% (≤55), and MD/PD gradient index (≤1.8) (HR: 0.30, *P* < 0.001 and HR: 0.36, *P* = 0.001) were identified as independent predictors of high risk of local tumor progression. Of note, type of SRS treatment and prior WBRT had no impact on tumor progression (Table 3).

**Table 3:** Multivariate analysis for local tumor progression and radiation necrosis (Per lesion analysis adjusted by the patient and SRS treatment)

Factors for local tumor progression	Hazard ratio for LP (95% CI)	*P*
Age	0.99 (0.98,1.01)	0.44
Systemic metastasis (no vs. yes)	0.95 (0.65,1.39)	0.79
New systemic progression at SRS (no vs. yes)	1.22 (0.84,1.78)	0.31
New ECM at SRS (no vs. yes)	0.47 (0.31,0.71)	***<0.001***
Neurological symptoms (none vs. mild vs. moderate, severe)	0.93 (0.74,1.17)	0.54
Post‐SRS hemorrhage (No vs. Yes)	0.78 (0.34,1.82)	0.57
Response to SRS		
CR vs. PR	2.20 (1.21, 3.95)	***0.009***
PR vs. Stable	1.23 (0.85, 1.78)	0.26
Stable vs. progression	10.2 (6.00, 17.4)	***<0.001***
Prior SRS to the other lesion (No vs. Yes)	0.91 (0.61,1.35)	0.62
Prior SRS to the same lesion (No vs. Yes) 1	11.96 (7.97,17.9)	***<0.001***
Volume (cc) (≤2.4 vs. >2.4)	1.05 (0.69,1.60)	0.82
Max tumor diameter (≤0.9 vs. >0.9)	2.31 (1.59,3.36)	***<0.001***
PD (≤22 vs. >22)	0.76 (0.48,1.21)	0.25
IDL% (≤55 vs. >55)	0.30 (0.15,0.59)	***<0.001***
Conformity (≤1.9 vs. >1.9)	0.98 (0.72,1.33)	0.90
Max dose (≤40 vs. >40) 2	0.79 (0.49,1.27)	0.33
MD/PD index (≤1.8 vs. >1.8)	0.36 (0.19,0.67)	***0.001***
*Factors for RN*	Odds Ratio for RN (95% CI)	*P*
Systemic metastasis (no vs. yes)	0.66 (0.46,0.96)	***0.029***
New systemic progression at SRS (no vs. yes)	0.93 (0.60,1.43)	0.73
Neurological symptoms (none vs. mild vs. moderate, severe)	1.04 (0.75,1.45)	0.80
1st f/u Neurological symptoms	0.40 (0.29,0.55)	***<0.001***
Response to SRS		
CR vs. PR	6.33 (2.83, 14.7)	***<0.001***
PR vs. stable	1.33 (0.90, 1.95)	0.14
Stable vs. progression	9.18 (5.22,16.1)	***<0.001***
Prior SRS to the other lesion (No vs. Yes)	0.60 (0.39,0.93)	***0.024***
Volume (cc) (≤2.4 vs. >2.4)	0.97 (0.59,1.61)	0.92
Max tumor diameter (≤ 0.9 vs. > 0.9)	1.84 (1.23,2.75)	***0.003***
IDL% (≤55 vs. >55)	0.98 (0.31,3.11)	0.97
Conformity (≤1.9 vs. >1.9)	0.62 (0.43,0.89)	***0.009***
Max dose (≤45 vs. >45) 2	1.04 (0.69,1.56)	0.84
MD/PD index (≤1.8 vs. >1.8)	1.36 (0.43,4.26)	0.60

SRS, stereotactic radiosurgery; ECM, extra cranial metastases; CR, complete resolution; PR, partial resolution; LP, local tumor progression; RN, radiation necrosis; IDL, Isodose line; PD, prescription dose; IDL, isodose line; MD/PD, mean dose/prescription dose.

values in italics means significant , *p*<0.05

### Prognostic Groups for local progression based on per lesion

**Figure 1:**
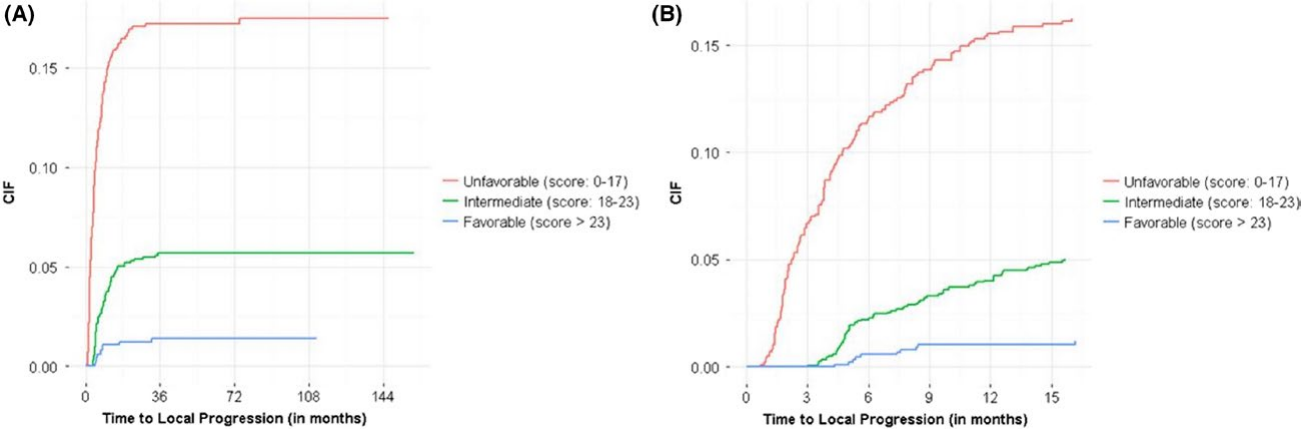
showing cumulative incidence of local tumor progression in patients with unfavorable (red), intermediate (green) and favorable (blue) prognostic scores at overall follow‐up (A) and at first 15 months follow‐up (B).

Independent prognostic factors (new ECM at SRS, response to SRS, prior SRS to the same lesions, tumor volume, IDL% and MD/PD gradient index) were identified based on competing risk stepwise selection model (Table 4). Based on the score derived from this model (range 0–27), three prognostic groups were identified (unfavorable: score 0–17; intermediate: score 18–23 and favorable: score 23–27). Patients in the favorable group had cumulative incidence (CIF) of local progression rate of 0.3%, 0.7% and 0.11% at 3, 6 and 12 months compared to 4.6%, 10% and 14.9% for patients in the unfavorable group respectively (HR: 0.25, 95% CI: 0.13, 0.47) (Table 4 and 5 and Fig. 1).

**Table 4:** Competing risk stepwise selection for local progression and radiation necrosis with prognostic group scores

Factor	Hazard/odds ratio (95% CI)	*P*‐value	Parameter estimate	Score	Factor levels and score
Local progression					
New ECM at SRS (no vs. Yes)	0.54 (0.39,0.74)	<0.001	−0.65	1	Yes: 1, no: 0
Response to SRS (CR vs. PR vs. S vs. P)	2.79 (2.24,3.49)	<0.001	1.04	2	CR: 15; PR: 10; S: 5; P: 0
Prior SRS to the same lesion (No vs. Yes)	11.05 (6.57,18.6)	<0.001	2.31	5	No: 5, yes: 0
Volume (cc) (≤2.4 vs. >2.4)	2.01 (1.48,2.73)	<0.001	0.98	2	≤2.4:2; >2.4:0
IDL% (≤55 vs. >55)	0.30 (0.15,0.60)	<0.001	−1.04	2	>55:2; ≤55:0
MD/PD gradient index (≤1.8 vs. >1.8)	0.38 (0.19,0.75)	0.005	−0.94	2	>1.8:2;≤1.8:0
Radiation necrosis					
Systemic metastasis (no vs. yes)	0.69 (0.50, 0.97)	0.032	−0.36	2	Yes: 2, no: 0
1st f/u Neurological symptoms	0.42 (0.33, 0.54)	<0.001	−0.86	5	Severe: 10; moderate:5 Mild: 0
Volume (cc) (≤2.4 vs. >2.4)	1.80 (1.22, 2.65)	0.002	0.59	4	≤2.4:4; >2.4:0
Response to SRS(CR vs. PR vs. S vs. P)	2.92 (2.40, 3.56)	<0.001	1.07	6	CR: 18; PR: 12; S: 6; P: 0

SRS, stereotactic radiosurgery; ECM, extra cranial metastases; CR, complete resolution; PR, partial resolution; S, stable; P, progression; LP, local tumor progression; RN, radiation necrosis; IDL, Isodose line; MD/PD, Mean dose/prescription dose.

values in italics means significant , *p*<0.05

**Table 5:** Prognostic groups for local tumor progression and radiation necrosis

Factor	No of patients (*n*)	3 months rate (95% CI)	6 months Rate (95% CI)	12 months rate (95% CI)	Hazard ratio (95% CI)	*P*‐value
Local tumor progression						
Unfavorable (score: 0–17)	831 (24%)	4.6% (3.6%, 5.8%)	10.0% (8.5%, 12.4%)	14.9% (12.4%, 17.9%)	–	
Intermediate (score: 18–23)	1805 (53%)	1.3% (0.9%, 1.7%)	2.8% (2.1%, 3.6%)	4.2% (3.3%, 5.4%)	0.26 (0.20, 0.36)	***<0.001***
Favorable (score > 23)	796 (23%)	0.3% (0.15%, 0.6%)	0.7% (0.0.4%, 1.2%)	0.11% (0.6%, 1.9%)	0.25 (0.13, 0.47)	***<0.001***

Factor	No of patients (*n* )	No. of necrosis	Odds Ratio (95% CI)	*P*‐value
Radiation necrosis				
Unfavorable (score: 0–16)	913 (26.7%)	151 (16.5%)	–	
Intermediate (score: 17–23)	1489 (43.6%)	84 (5.6%)	0.30 (0.22, 0.41)	***<0.001***
Favorable (score > 24)	1009 (29.6%)	8(0.79%)	0.14 (0.06, 0.29)	***<0.001***

values in italics means significant , *p*<0.05

### Univariate and multivariate analysis for RN

Overall, the incidence of RN based on per lesion was 5.7% (*n* = 245) in our series. Univariate analysis adjusted by patient and SRS treatment showed various patient (absence of systemic metastasis, absence of new systemic metastasis or progression at SRS, good neurological function at SRS, good neurological status at 1st follow‐up and tumor progression following SRS) and lesion (no prior SRS to the other lesion, large tumor volume, large maximum target diameter, high maximum dose, low IDL, low conformity, and MD/PD gradient index) related factors were associated with a higher risk of RN following SRS (Table 3).

On multivariate analysis, absence of systemic metastases (HR: 0.66, *P* = 0.029), good neurological function at 1st follow‐up (HR: 0.40, *P *≤ 0.001), response to SRS at first follow‐up MRI (partial resolution of lesion compared to complete resolution [HR: 6.33, *P* < 0.001], progression of tumor compared to stable size following SRS [HR: 9.18, *P* < 0.001]), no prior SRS to other lesion (HR: 0.60, *P* = 0.024), large maximum target diameter (>0.9 cm) )HR: 1.84, *P* = 0.003) and conformity index (≤1.9) (HR: 0.62, *P* = 0.009) and were independent predictors of high risk of RN (Table 3). Tumor volume had no impact on the incidence of tumor necrosis. Interestingly, patients with severe neurological symptoms at first follow up were less likely to develop RN, likely as they did not live long enough to develop this complication and were identified as favorable prognostic factors, therefore this finding needs to be interpreted cautiously. Also, there was no significant difference in patients with stable or partial response to SRS and were assigned similar scores in the prognostic model.

### Prognostic groups for RN

Independent prognostic factors (absence of systemic metastases, 1st neurological symptoms, tumor volume and response to SRS) were identified based on marginal logistic regression stepwise selection model Table 4. Although tumor volume was not significant in the multivariate model, we included this factor in the prognostic model, as it is clinically relevant and useful in decision making.

Based on scores derived from this model (range: 0–34), four prognostic groups were identified (unfavorable: score 0–16; intermediate: score 17–23 and favorable: score 24–34). Patients in the unfavorable group had higher incidence of RN compared to those in favorable (16.5% vs. 0.79%, *P* < 0.001), and intermediate group (16.5% vs. 5.6%, *P* < 0.001) (Table 4 and 5).

## Discussion

This is the first study aimed to identify high‐risk patient population for local tumor progression and RN based on first follow‐up radiographic response following SRS for BM. We have created prognostic groups using marginal logistic regression stepwise selection model.

EORTC (22952‐26001) 40 and other randomized studies 1, 2 have shown that adjunct WBRT following surgery or SRS has no impact on overall survival in patients with a limited number (1–4) of BMs. However, there is a low‐quality evidence suggesting adding upfront WBRT to surgery or SRS results in a decrease in intracranial tumor progression at 1 year by 53% (RR: 0.47, 95% CI: 0.34–0.66, *P* < 0.0001) 1.Various factors such as age^20^, performance status 20, status of systemic disease 20, 41, tumor volume 25, tumor histology 20, 42, 43, location 25, 44, number of BM 20, radiation dose 20, 41, 43, intratumoral hemorrhage 42 and adjunct WBRT 1, 2 have been identified as predictors of local tumor progression and overall outcomes (neurological/nonneurological) following SRS for BM 20, 27, 43, 44, 45, 46. Similarly, factors such as prior SRS, primary tumor histology (renal), connective tissue disorder and systemic therapy (capecitabine) have been identified as risk factors for AREs following SRS for BMs 21. Despite the availability of these pre‐SRS predictive factors, there is a lack of evidence suggesting the correlation between post‐SRS response and outcome.

### Factors predictive of local tumor progression

In our study, absence of new ECM at SRS and large maximum tumor diameter were found to be independently associated with increased risk of local tumor progression. McTyre et al. 20 in their series of 738 patients with BM treated with upfront SRS without WBRT, identified histology (melanoma), DS‐GPA, number of BM and SRS dose as predictors of neurological death. Our results showed that complete resolution of BM with SRS at first follow‐up MRI was a good prognostic factor (HR: 2.20, *P* = 0.009), whereas progression of the BM was a poor prognostic factor (HR: 10.2, *P* < 0.001). Interestingly, stable disease or partial response to SRS had same impact on local progression (HR: 1.23, *P* = 0.26). Based on the prognostic groups with highest impact of response to SRS on the scores, patients in the favorable score had 0.7% risk of tumor progression at 6 months compared to 10% for those in the unfavorable group. This finding has important implications in the sense that patients with stable or partial response to SRS need a closer follow‐up and monitoring, whereas those with complete response can have a longer follow‐up if they are stable for first few follow‐ups. Also, we found that presence of new ECM at SRS is a favorable prognostic factor whereas systemic disease progression has no effect on local tumor progression which is in contrast to that reported McTyre et al. 20. This difference may be attributed to the fact that the end point in our study was local tumor progression (compared to overall survival in the other study 20) and patients with progressive systemic disease or with new ECM did not live long enough to develop local tumor progression. In addition, patients with new ECM at SRS might be started on active chemotherapy for their progressive systemic disease and likely be benefitted in terms of local tumor control based upon CNS penetration of their systemic treatment.

Smaller intracranial volume (≤2.4 cc), higher IDL (>55%) and MD/PD gradient index (>1.8) were identified as favorable prognostic factors in our study. Our findings are concordant with Romano et al. 47, who reported that patients with smaller tumor volume treated at higher IDL had superior local tumor control rates. Gradual dose fall off at the tumor margin associated with higher IDL has been shown to control the microscopic metastatic disease beyond the visible tumor margins on imaging 47, 48, 49.

### Factors predictive of RN

The incidence of RN in our series was 5.7% which is within the range of 5–24% in previously reported series 21, 22, 23, 24, 25. Similar to local tumor progression, response to SRS was identified as a strong independent predictor of RN, with complete response being a favorable factor for not developing RN, progression as unfavorable and partial response/stable as an intermediate predictor. Therefore, tumor response to SRS at first follow‐up MRI can be used to predict the likelihood of both local tumor progression (reduced risk) and RN (increased risk). Tumor volume and maximum tumor diameter (>0.9 cm) had an impact on RN on univariate analysis (HR: 1.94 and 3.27 respectively, *P* < 0.001) and large maximum tumor diameter had an impact on multivariate analysis as well (HR: 1.84, *P* = 0.003). Kilburn et al.^25^ reported that increasing tumor volume was correlated with increased risk of toxicity (HR: 1.63 per cc, *P* = 0.01, 95% CI: 1.11–2.40) in patients with brainstem metastases. The overall incidence of RN in this series was 9.09% and 40% of patients with tumor volume > 1 cc developed grade 2–3 toxicity. They identified tumor volume >1 cc as a predictor of treatment‐related toxicity, as reported by Kased et al. 50 too. Given that tumor volume has an impact on RN, we have included this factor in the marginal logistic stepwise selection for creating prognostic groups for RN (despite not being significant on multivariate analysis) and found that patients with smaller tumor volume (≤2.4 cc) is a favorable prognostic factor. Of note, presence of systemic metastasis and 1st follow‐up poor neurological function was identified as favorable prognostic factors for not developing RN. This finding needs to be carefully interpreted as these patients were likely to have not live long enough to develop RN, which may take few months to years to develop 22. In our recent study, tumor histology (renal and lung adenocarcinoma) and molecular markers (HER2 amplification and ALK/BRAF mutation) were also identified as risk factors for RN following SRS for BM 23.

Low conformity index and no prior SRS to other lesion were also identified as independent risk factors for developing RN following SRS [HR: 0.62, *P* = 0.009 and HR: 0.60, *P* = 0.024, respectively]. Valery et al. 51 identified conformity index as the only parameter affecting the risk of RN following LINAC radiosurgery for BM in 377 patients (760 BMs). These factors point toward reducing the dose of radiation to the normal brain tissue to decrease the risk of RN. However, as mentioned in the above section on local tumor control, studies have reported that including a margin of normal brain tissue (1–2 mm) results in superior local tumor control 47, 48, 49, 52, therefore it is prudent to create a *“just perfect”* plan to achieve good tumor control and avoid RN at the same time.

### Strengths and limitations

The retrospective nature and the use of heterogeneous tumor types are potential limitations of our study. However, our study is the first one to demonstrate the association between first radiographic response and tumor progression or development of RN in a large sample size. We have also identified prognostic groups based on various factors which can be incorporated into routine clinical practice.

## Conclusions

Complete resolution of tumor at first follow‐up is a good prognostic factor for both local progression and development of RN after SRS. In addition, presence of new extra cranial metastasis at SRS, tumor size <0.9 cm/tumor volume <2.4 cc and no prior SRS to the index lesion are other good prognostic factors with reduced risk of local tumor progression following SRS. Regarding RN, other than complete response to SRS, poor neurological function at first follow‐up, prior SRS to other lesions and high conformity index (>1.9) are favorable factors for not developing RN. Stable or partial response at first follow‐up after SRS have same impact on local progression and RN compared to those with complete resolution or progression. These factors can be considered to formulate appropriate follow‐up while managing these patients.

## Conflict of Interest

None declared.
